# The DNA damage tolerance factor Rad5 and telomere replication

**DOI:** 10.1007/s00294-025-01315-y

**Published:** 2025-05-26

**Authors:** Stefano Mattarocci

**Affiliations:** Université Paris-Saclay, Université Paris-Cité, CEA, Institut de biologie François Jacob, UMR Stabilité Génétique Cellules Souches et Radiations, Fontenay-aux-Roses, Inserm France

**Keywords:** DNA replication, Telomere, DNA repair pathways, Rad5, Cell fate, ALT

## Abstract

The DNA Damage Tolerance pathway (DDT) is one of the major mechanisms for resolving replication fork blocks. A key factor in DDT is the fork-associated clamp PCNA, which can undergo to mono- or polyubiquitination, leading to error-prone or error-free modes of DNA damage bypass, respectively. In the yeast *Saccharomyces cerevisiae*, Rad5^HLTF/SNF2^ factor plays important roles in both pathways: (i) promoting the error-free mode through PCNA polyubiquitination and transient template switching and (ii) interacting with specialized DNA polymerases involved in the error-prone pathway. Rad5 also associates with telomeres, the repetitive DNA regions present at the ends of chromosomes. Telomeric DNA, tightly bound by tandem proteins arrays, poses unique challenges to replication fork progression. Here, I review the current understanding of the link between Rad5 and telomeres and provide evidence that Rad5 binds to yeast telomeres, with notable enrichment during telomere replication. This finding highlights a connection between telomeres and an important DDT factor in unperturbed wild-type cells, raising intriguing possibilities regarding the functional interplay between telomere replication and DNA damage tolerance mechanisms.

**Supplementary Information:**

The online version contains supplementary material available at 10.1007/s00294-025-01315-y.

## Rad5^HLTF/SNF2^: a central factor for the DDT pathway

The DNA replication process ensures that the genome is fully duplicated prior to cell division. However, replication can be challenged by a multitude of endogenous and exogenous stresses. Endogenous stresses arise from natural barriers within the DNA sequence, such as tightly packed chromatin, repetitive sequences, DNA secondary structures, or DNA damages caused by reactive oxygen species (Saxena and Zou 2022). These obstacles can impede replication fork progression (Saxena and Zou 2022). Similarly, exogenous factors, such as exposure to UV or chemical agents, induce DNA lesions that, if not repaired, can also challenge replication (Kciuk et al. 2020). Both endogenous and exogenous stresses can lead to stalled replication forks. Unresolved stalled forks can result in the collapse of the replication machinery, resulting in DNA breakage and compromising overall genome stability. To counteract these threats, cells have evolved dedicated pathways, including the DNA damage tolerance pathway (DDT), also referred as post-replication repair pathway or Rad6/Rad18-dependent DNA damage tolerance pathway (see below). This evolutionary conserved pathway plays a role in bypassing DNA lesions that would otherwise block replication fork progression, thereby preventing fork collapse and maintaining genome stability (Ulrich 2011; Branzei and Szakal 2016; Arbel et al. 2020, 2021). The key player of the DDT pathway is PCNA, a sliding homotrimeric clamp for DNA polymerases and an essential component of the replication machinery (Moldovan et al. 2007; Arbel et al. 2020, 2021; Bellí et al. 2022).

Stalled DNA replication forks frequently result in polymerase-helicase uncoupling as well as DNA resection, leading to excessive production and consequent accumulation of single-stranded DNA (ssDNA) (Liao et al. 2018; Técher and Pasero 2021). This ssDNA covered by RPA leads to an activation of the S-phase checkpoint. In *Saccharomyces cerevisiae*, the RPA-ssDNA filament recruits Rad18, an E3 ubiquitin ligase, which engages Rad6, an E2 ubiquitin conjugase, to form the Rad6/Rad18 complex. This complex translocates along RPA filaments and monoubiquitinates PCNA at lysine 164 (Liefshitz et al. 1998; Hoege et al. 2002; Li et al. 2020). This step is crucial to allow the recruitment of translesion-synthesis (TLS) DNA polymerases (in yeast: Rev1, Pol ζ (Rev3-Rev7-Pol31-Pol32), Pol η (Rad30). These error-prone polymerases are less hindered by DNA lesions, therefore allowing replication through the lesions (DDT error-prone bypass pathway) (Prakash et al. 2005). On the other hand, in yeast cells, PCNA_K164_Ub (but not the unmodified PCNA) can be polyubiquitinated at lysine 63 by the E2 ubiquitin conjugase Ubc13-Mms2 and the E3 ubiquitin ligase Rad5 (Ulrich 2001; Hoege et al. 2002; Torres-Ramos et al. 2002; Xu et al. 2015). This modification activates the error-free DDT pathway, which relies on a transient template-switching (TS) mechanism where the stalled DNA strand utilizes the newly synthesized sister chromatid strand as a template to bypass the lesion (Minca and Kowalski 2010; Giannattasio et al. 2014).

**Fig. 1:**
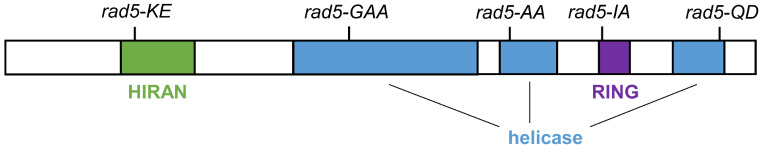
Schematic representation of Rad5 domain structures. The domain structures of Rad5 are depicted, highlighting the HIRAN domain (in green), the helicase domain (in blue), and the RING ubiquitin ligase domain (in violet). For each domain, known *rad5* alleles harboring specific point mutations are shown. The *rad5-AA* allele, containing the D681A and E682A mutations, the *rad5-GAA* allele (K538A and T539A mutations), and the *rad5-QD* allele (Q1106D mutation), all within the helicase domain, are depicted (Choi et al. 2015; Ball et al. 2014; Gallo et al. 2019; Gangavarapu et al. 2006; Ortiz-Bazan et al. 2014). The *rad5-IA* allele (I916A mutation), a point mutation in the ubiquitin ligase domain, disrupts the Rad5-Ubc13 interaction and, consequently, this allele is defective for template switching repair activity (Ulrich 2003; Carlile et al. 2009; Toth et al. 2022; Jiang et al. 2023). The *rad5-KE* allele (K194E mutation), located in the HIRAN domain, impairs fork regression activity (Shin et al. 2018; Gallo et al. 2019)

Yeast Rad5 belongs to the SWI/SNF2 family of ATPases and contains seven conserved helicase-like motifs, a Rad5 enzymatic activity important for the TS pathway (Fig. 1) (Johnson et al. 1992, 1994; Ball et al. 2014). Rad5 contains also a RING finger domain, characteristic of the ubiquitin ligase enzymes, which is inserted between the helicase motifs III and IV (Lorick et al. 1999; Unk et al. 2010). Additionally, the N-terminal region of Rad5 harbors a HIRAN domain, proposed to function as DNA-binding domain (Iyer et al. 2006; Fan et al. 2018) (Fig. 1). Two homologs of *S. cerevisiae* Rad5 have been identified in mammalian cells, playing similar roles in the TS pathway: the “Helicase-Like Transcription Factor” HLTF and the “SNF2 Histone linker PHD RING Helicase” SHPRH (Unk et al. 2010; Chavez et al. 2018; Blastyak et al. 2010). These three proteins are members of the SWI/SNF2 family of ATP-dependent DNA translocases involved in chromatin remodeling and DNA repair, sharing an ubiquitin ligase RING motif (Unk et al. 2010). HLTF and Rad5 also share the HIRAN domain (Iyer et al. 2006; Kile et al. 2015; Korzhnev et al. 2016).

Rad5 interacts with Rad18 and PCNA, and both interactions are important for its localization at sites of DNA damage (Ulrich and Jentsch 2000; Moldovan et al. 2007; Carlile et al. 2009). Recent study reported that Rad5 shuttles between nuclear regions under replication stress, accumulating in foci where the DDT pathway is active (Lehmann et al. 2024). The greater UV-sensitivity of cells lacking Rad5 compared to cells lacking Mms2 or Ubc13 suggests that Rad5 could play additional roles beyond the activation of the TS pathway (Gangavarapu et al. 2006). Rad5 has been shown to interact with Rev1, one of the TLS polymerases (Pagès et al. 2008; Kuang et al. 2013; Xu et al. 2016). Moreover, using a Rad5 mutant defective in template switching repair activity (*rad5-IA*; Fig. 1) (Ulrich 2003; Carlile et al. 2009; Toth et al. 2022), Jiang et al. identified two independent pathways differing in the recruitment of Rev1—and consequently other TLS polymerases—to the site of action. In one pathway, recruitment occurs directly via PCNA, while in the other, Rad5 also facilitates the PCNA-dependent process by acting as a scaffold protein (Jiang et al. 2023). Based on these data, Rad5 could play a role in the choice between error-prone and error-free DDT pathways (Pagès et al. 2008; Xu et al. 2016; Ortiz-Bazán et al. 2014; Choi et al. 2015; Gallo et al. 2019).

In vitro study indicates that yeast Rad5 and mammalian HLTF can bind various DNA structures (Blastyák et al. 2007) and possess DNA helicase activity specific for replication fork reversal (Shin et al. 2018; Ling et al. 2023), both activities mediated by the HIRAN domain (Kile et al. 2015; Shin et al. 2018; Ling et al. 2023; Shen et al. 2021). Furthermore, overexpression of *RAD5* seems to lead to an accumulation of fork reversal-like intermediates in vivo (Bryant et al. 2019). Rad5 and HLTF can regress replication fork to form “chicken foot” structures, which are thought to be important for the stabilization and the rescue of stalled replication forks (Shin et al. 2018; Bai et al. 2020; Shen et al. 2021). Fork reversal can rescue DNA replication after a block by promoting the DDT pathway or by limiting the uncoupling of leading and lagging DNA strand synthesis, thereby preventing the accumulation of single-stranded DNA (Neelsen and Lopes 2015; Berti and Vindigni 2016). Overall, these data suggest that Rad5 contributes to DNA lesion bypass through multiple pathways: (i) strand invasion-dependent template switching (TS), mediated by its translocase and ubiquitin ligase activities; (ii) the promotion of TLS polymerase activity, which is likely independent Rad5’s enzymatic functions; and (iii) replication fork reversal mediated by its HIRAN domain (Toth et al. 2022).

### Telomeres: a challenging region for DNA replication

Specific genomic regions can challenge the DNA replication machinery. Among these, telomeres - the nucleoprotein structures capping the ends of chromosomes - pose problems for the replication process even in absence of exogenous replication stress (Mason-Osann et al. 2019; Cicconi and Chang 2020; Stroik and Hendrickson 2020; Bonnell et al. 2021). Telomeres are composed of G-rich repetitive sequences tightly bound in tandem by specialized proteins that protect the terminal portions of chromosomes (Longhese et al. 2012; De Lange 2018). These G-rich sequences could form secondary structures, such as G-quadruplex (Traczyk et al. 2021; Xu and Komiyama 2023), which are known to impede replication fork progression (Joo et al. 2024). Stabilizing these structures with chemical compounds reduce cell viability, possibly due to telomere replication failure (Vertecchi et al. 2022). Moreover, telomeres are transcribed into non-coding RNA known as TERRA, which could form R-loops, i.e. DNA-RNA hybrids that could impede fork progression (Rivosecchi et al. 2024; In et al. 2025).

Another important source of replication obstacles at telomeres is represented by the proteins that directly bind to the telomeric repeats: Rap1 in budding yeast, Taz1 in fission yeast and TRF1-TRF2 in mammals (König et al. 1996; Cooper et al. 1997; Kupiec 2014; De Lange 2018; Bonetti et al. 2020). Despite structural differences, Rap1, Taz1 and TRF1/2 utilize conserved Myb-like motifs to bind telomeric DNA (König et al. 1996; Cooper et al. 1997; Matot et al. 2012; Chen 2019). *S. cerevisiae* Rap1’s tight binding to telomeric repeats (Williams et al. 2010; Analikwu et al. 2023) represents a significant block to replication fork progression (Douglas and Diffley 2021). Rap1 also impede other DNA transactions, including transcription (Wu et al. 2018; Challal et al. 2018) and condensin loop extrusion (Analikwu et al. 2023). While tight DNA-protein binding is essential for efficient telomere end protection (Mattarocci et al. 2024), it may come at a cost, creating challenges for specific DNA transactions, including DNA replication.

Several evidence point out that telomere regions could be “hot-spots” for the accumulation of replication intermediates, which in turn recruit various DNA repair factors, including checkpoint proteins, helicases and the Rad51 recombinase (Brenner and Nandakumar 2022; Maestroni et al. 2017; Higa et al. 2017). Several proteins help the replication machinery in passing through telomeres (e.g.: the Pif1 and Rrm3 helicases) (Makovets et al. 2004; Geronimo and Zakian 2016). Telomerase - the holoenzyme elongating short telomeres - can also be considered a repair factor, since it repairs broken telomeres resulting from replication defects by extending them (Matmati et al. 2020).

In the absence of telomerase, other compensatory pathways become essential for telomere stability. As cells divide, telomeres progressively shorten, eventually to a point where they become dysfunctional and prone to fusion or extensive resection (Teixeira 2013; Pobiega et al. 2021). This uncapping/de-protection of telomeres triggers a persistent Mec1^ATR^-dependent checkpoint response, ultimately leading to cell death, an outcome termed “telomere crisis” (Lundblad and Szostak 1989). Critically short telomeres whose replication has been defective could be viewed as dysfunctional and in need to be repaired. Consequently, the survival and proliferation of telomerase-negative cells depends on the activation of repair pathways and thus of the recruitment of repair factors at chromosome ends. Rad5 has been proposed to be involved in this response (see below; Fallet et al. 2014).

### Does Rad5 play a role in telomere replication?

The link between Rad5 and telomere biology was discovered serendipitously. In budding yeast, it has been proposed that artificially shortening a single telomere accelerates the telomere crisis caused by the loss of telomerase (Abdallah et al. 2009). Notably, the W303 genetic background used in that initial study, carries a mutant allele of the *RAD5* gene, *rad5-535*. This allele harbors a point mutation in one of the seven consensus motifs of the Rad5 helicase domain (Fan et al. 1996). Cells with the *rad5-535* mutation exhibit defects in the error-prone TLS pathway, resulting in heightened sensitivity to DNA-damaging agents such as MMS and increased genomic instability (for more details, see *“Choose your yeast strain carefully: the RAD5 gene matters”* (Elserafy and El-Khamisy 2018). By assessing the relevance of this Rad5 partial defect in the response to telomere shortening, subsequent work showed that the absence of Rad5 greatly exacerbates the cell death caused by an artificially short telomere in telomerase-negative cells (Fallet et al. 2014). Interestingly, another DDT pathway factor, Mms2, was also found to be involved in the maintenance of short telomeres in the absence of telomerase (Fallet et al. 2014). The discovery that Rad5 and the DDT pathway have a role at telomeres is intriguing, as it directly links telomere replication to factors that assist DNA replication under stress. Supporting this idea, Rad5 binds telomeres in telomerase-positive cells (Fallet et al. 2014). Despite these initial hints, direct evidence of Rad5’s implication in telomere replication remained unclear. Thus, I investigated whether Rad5 could be enriched at telomeres during their replication.

In budding yeast, there is a temporal program for DNA replication, where specific origins fire early in S phase (early origins) and others later (late origins). Telomeres are among the last regions to replicate (McCarroll and Fangman 1988; Raghuraman et al. 2001; Theulot et al. 2025). To investigate whether Rad5 associates with replicating telomeres, I used Chromatin Immunoprecipitation (ChIP) coupled with cell cycle synchronization during S phase (Bianchi and Shore 2007; Mattarocci et al. 2014; Hafner et al. 2018). Telomerase-positive cells were arrested in G1 and subsequently released at a low temperature (18.5 °C) to slow down the replication fork progression, thereby improving the temporal resolution between early and late origins.

**Fig. 2:**
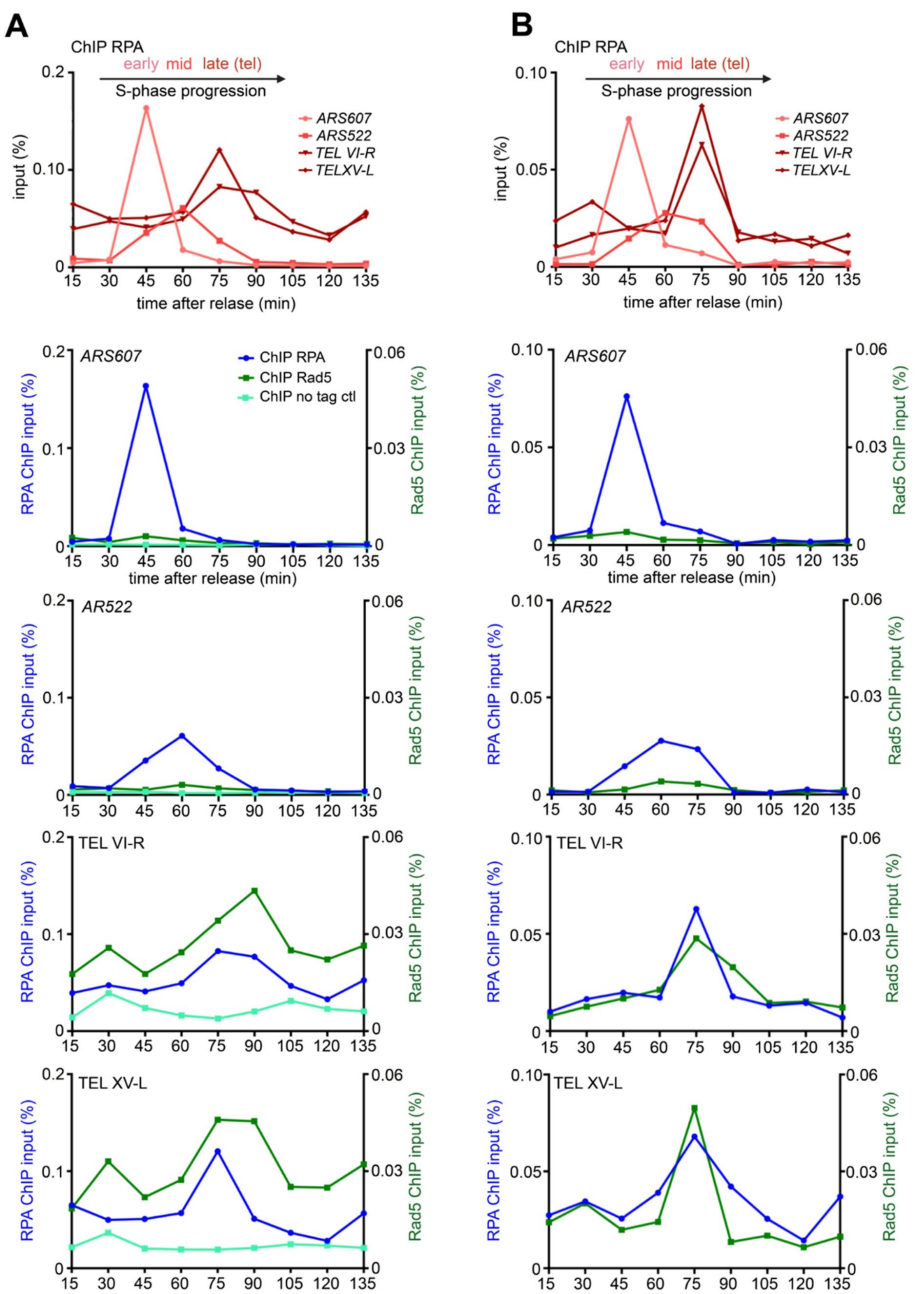
Rad5 is enriched at replicating telomeres. **A**-**B** (upper graphs). Two biological replicates of RPA ChIP-qPCR experiments performed in Rad5-Myc tagged cells. The cells synchronization and release was performed as previously described (Bianchi and Shore 2007; Mattarocci et al. 2014; Hafner et al. 2018). Exponential growing cells were blocked in G1 by the addition of α-factor (for 130 minutes, final concentration: 10^− 8^ M) and subsequently released at 18.5°C by washing away and degrading the α-factor by Pronase addition (56 mg in 540 ml of YPD media; Millipore 537088R). Cell-cycle synchrony and progression were monitored by microscopy. Following release from the G1 block, samples were collected at 15-minute intervals for ChIP analysis (time point 0 min corresponds to Pronase addition). ChIP was performed has previously described (Mattarocci et al. 2014; Shyian et al. 2016; Hafner et al. 2018; Mattarocci et al. 2024). RPA enrichment (primary Ab used: anti-RPA, polyclonal Agrisera AS07214) at specific genomic loci was assessed: *ARS607* (early replicative origin, in light red), *ARS522* (mid/late replicative origin, in red), and subtelomeric regions TEL VI-R and TEL XV-L (in dark red, qPCR edge approximately 100 bp from the telomeric TG_1 − 3_ repeat). Results are shown as a percentage of the input fraction. **A-B** (bottom graphs). Half of the samples processed for RPA ChIP (upper panels) were used for ChIP of Rad5-Myc (two replicates shown, in dark green). To facilitate the comparison, RPA enrichment curves (blue) from the upper panel are also displayed in the lower panels. In the left replicate (A), a no-tag control ChIP (light green curve) is included, using anti-Myc immunoprecipitation (primary Ab used: anti-Myc, Sigma-Aldrich 05-724) in untagged *RAD5* cells. The background signal detected at native telomeres in the untagged strain was relatively high throughout the cell cycle compared to non-telomeric loci but remained approximately 3-fold lower than the Rad5-Myc signal and did not increase at the time of telomere replication (75–90 min). The primers used for the qPCR step of the ChIP assay were as followed: TEL XV-L 5’-ATCGTGGTTCGCTGTGGTAT-3’ and 5’-AACCCTGTCCAACCTGTCTCC-3’; TEL VI-R 5’-TCCGAACTCAGTTACTATTGATGGAA-3’ and 5’-CGTATGCTAAAGTATATATTACTTCACTCCATT-3’; ARS607 5’-TCTGAACTGCAAATTTTTGTCATA-3’ and 5’-AGCCTTGTGCAGAAAGCATATGT-3’; ARS522 5’-CGTTCGAAAACCGGATATGT-3’ and 5’-CCCGATGACTACGAGGCTAT-3’; *OGG1* 5’-CAATGGTGTAGGCCCCAAAG-3’ and 5’-ACGATGCCATCCATGTGAAGT-3’. Strains are from a *RAD5*-corrected W303 background (genotype: *MATa bar1Δ ura3-1 trp1-1 leu2-3 112 his3-11 can1-100 RAD5 ADE2*)

To track replication fork progression, I monitored the recruitment of the single-stranded DNA binding protein RPA (Waga and Stillman 1998; Mattarocci et al. 2014; Hafner et al. 2018). As shown in Fig. 2A & B (top graphs) and Suppl. Figure 1, and consistently with previous reports (Mattarocci et al. 2014; Hafner et al. 2018), RPA was recruited to the early origin *ARS607* approximately 45 min after release from the G1 block, producing a sharp ChIP peak that reflect the high firing efficiency of early origins (Theulot et al. 2025). The mid/late origin *ARS522* was replicated predominantly at 60 min, with a broader peak indicative of its lower firing probability. As expected, (Bianchi and Shore 2007; Mattarocci et al. 2014), replication fork passage at two native telomeres (TEL VI-R and XV-L) occurred later, peaking between 75 and 90 min after release. Interestingly, RPA enrichment at telomeres was also higher throughout the cell cycle compared to its enrichment at internal origins.

The same ChIP samples analyzed for RPA recruitment were then assessed for the recruitment of Rad5, tagged with Myc epitopes (Fig. 2A & B, bottom graphs; Suppl. Figure 1). As a control, an untagged strain was included (Fig. 2A). At the two internal origins, *ARS607* and *AR522*, a slight Rad5 specific enrichment above the control at the time of their respective replication (45 min and 60–75 min respectively) was detected. This suggests that Rad5 may associate with the replication forks in a subset of cells. In contrast, at the two native telomeres (TEL VI-R and XV-L), Rad5 enrichment was consistently higher for all the time points analyzed compared to the internal regions and the no-tag control. Interestingly, Rad5 enrichment further increased during telomere replication (75–90 min), coinciding with the peak of RPA recruitment (Fig. 2A & B; Suppl. Figure 1). Overall, these findings suggest that Rad5 could be already present at telomeres before the passage of the replication fork and becomes further enriched during telomere replication in wild-type cells.

### Putative functions of Rad5 at telomeres

**Fig. 3:**
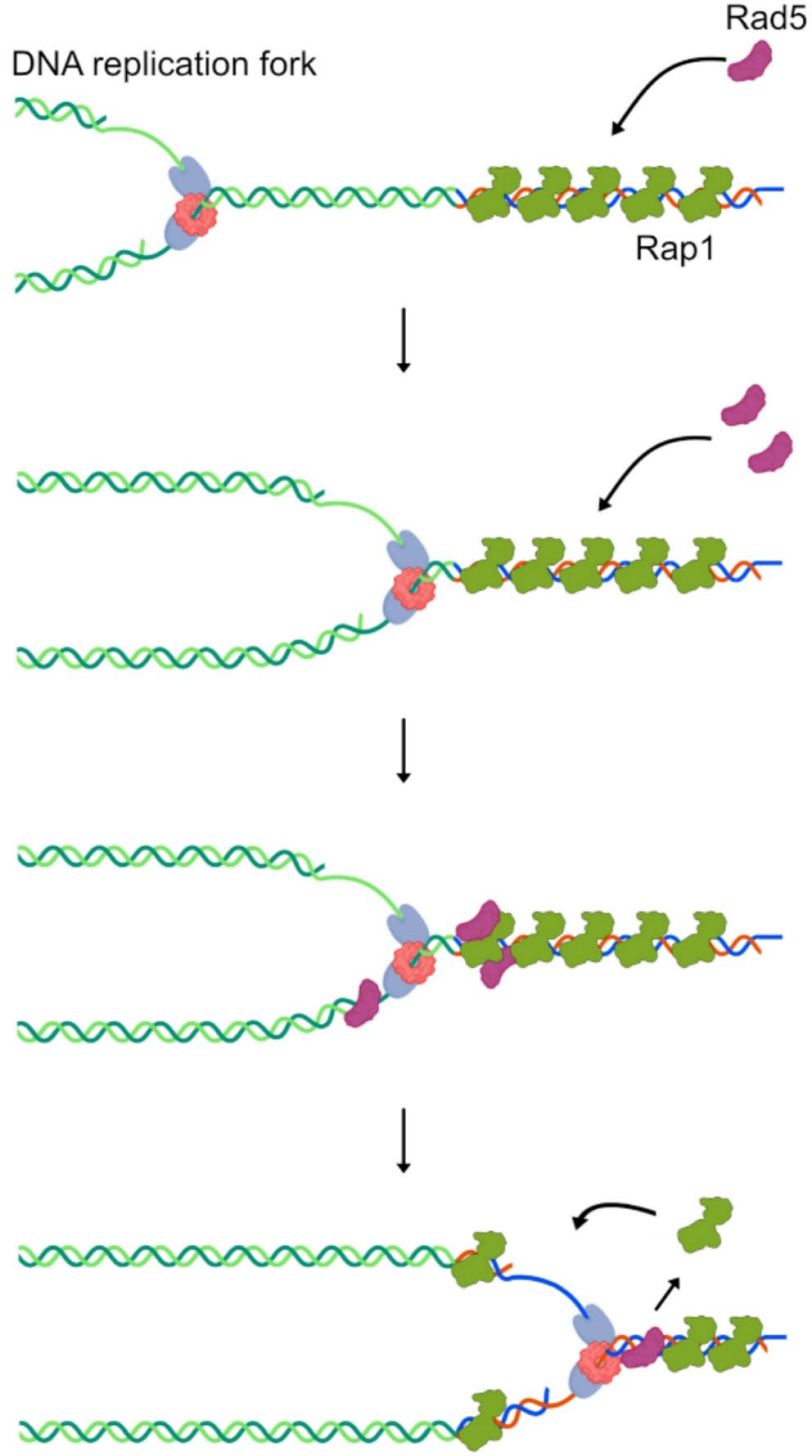
Scheme of possible Rad5 functions at native telomeres. A representative schematic of the DNA replication fork is shown with leading and lagging DNA polymerases depicted in light blue, and the MCM helicase complex in light red. Telomeric repeats are represented by DNA strands in blue and red, while the budding yeast telomere-binding protein Rap1 is shown in green. Analysis of Rad5 recruitment (Fig. 2) suggests that Rad5 (in dark red) is present at telomeres before the DNA replication fork reaches the telomeric repeats. Studies indicate that the DNA replication fork could transiently stall at telomeres, likely due to the presence of tightly bound proteins, such as Rap1 (Douglas et al. 2021), or secondary DNA structures (not shown in the scheme) (Traczyk et al. 2021; Joo et al. 2024; Rivosecchi et al. 2024). Fork stalling may promote further recruitment of Rad5, potentially facilitating fork resumption through an as-yet unidentified mechanism. The progression of the DNA replication fork is thought to progressively release Rap1 from telomeres, which subsequently recycles back once the double-stranded telomeric DNA is re-formed

This study describes a connection between the replication of native telomeres, a genomic region inherently challenging to replicate, and Rad5, a multifunctional protein that facilitates DNA replication fork progression under stress conditions. An intriguing hypothesis is that Rad5 may be required at telomeres to assist DNA fork replication even in absence of exogenous replication stresses. Understanding how Rad5 is recruited to telomeres remains a key question. One potential mechanism is that Rad5 may be recruited in two distinct steps. Initially, Rad5 may be directed to telomeres through interactions with other factors, such as proteins involved in the DNA damage tolerance (DDT) pathway or telomere-associated proteins. Subsequently, if the DNA replication fork stalls at telomeres, Rad5 may engage directly with DNA via its HIRAN domain, thereby facilitating fork restart and maintaining genomic integrity (Fig. 3).

The specific functions mediated by Rad5 at telomeres in telomerase-positive cells remains to be explored too. In telomerase-negative cells, Rad5 postpones telomere crisis, suggesting that Rad5-dependent template switching or fork reversal helps cells to cope with critically short telomeres (Fallet et al. 2014; Aguilera et al. 2020). Similarly, in telomerase-positive cells, Rad5-dependent template switching or fork reversal could help to rescue abnormally short telomeres that can arise sporadically even in the presence of telomerase. In this scenario, Rad5 could be constitutively recruited to telomeres in anticipation of these rare events. Alternatively, Rad5 and the DDT pathways may operate at all telomeres, not only at the shortest ones. For instance, template-switch or fork reversal could help the recovery of DNA replication forks that pause at Rap1-bound telomere repeats (Fig. 3). Rad5 may also play a non-enzymatic role at telomeres, acting as a platform for the recruitment of other factors (Jiang et al. 2023; Toth et al., 2022). Genetic analysis using specific point mutations in Rad5 domains (Fig. 1) will be instrumental in elucidating both Rad5 recruitment and the molecular mechanisms underlying its functions at telomeres. Additionally, further studies investigating the multiple functions of yeast Rad5 at telomeres are expected to provide key insights into the DDT pathways and telomere replication.

The DDT pathway and Rad5 functions are evolutionary conserved, as are helicases that act at telomeres to cope with replication stresses. Evidence supporting the involvement of the DDT pathway at mammalian telomeres comes from a recent study demonstrating that damaged human telomeres accumulate two DDT-related factors, Rad18 and the ubiquitinated form of PCNA (Zhang et al. 2023). This suggests the possibility that the link between DDT pathway — particularly involving Rad5 and its homologs — and telomere replication in unstressed cells may extended to other organisms.

## Electronic supplementary material

Below is the link to the electronic supplementary material.

Supplementary Material 1

## Data Availability

No datasets were generated or analysed during the current study.
