# Supplementary material for: The DNA damage tolerance factor Rad5 and telomere replication
Source: Curr Genet. 2025 May 26;71(1):11. doi: 10.1007/s00294-025-01315-y (PMC12106482; doi:10.1007/s00294-025-01315-y)
Supplement: Supplementary file 1 — Supplementary Material 1 [file 294_2025_1315_MOESM1_ESM.docx]

**Supplementary information**

**The DNA Damage Tolerance Factor Rad5 and Telomere Replication**

Stefano Mattarocci^1^

^1^Université Paris-Saclay, Université Paris-Cité, CEA, Inserm, Institut de biologie François Jacob, UMR Stabilité Génétique Cellules Souches et Radiations, Fontenay-aux-Roses, France


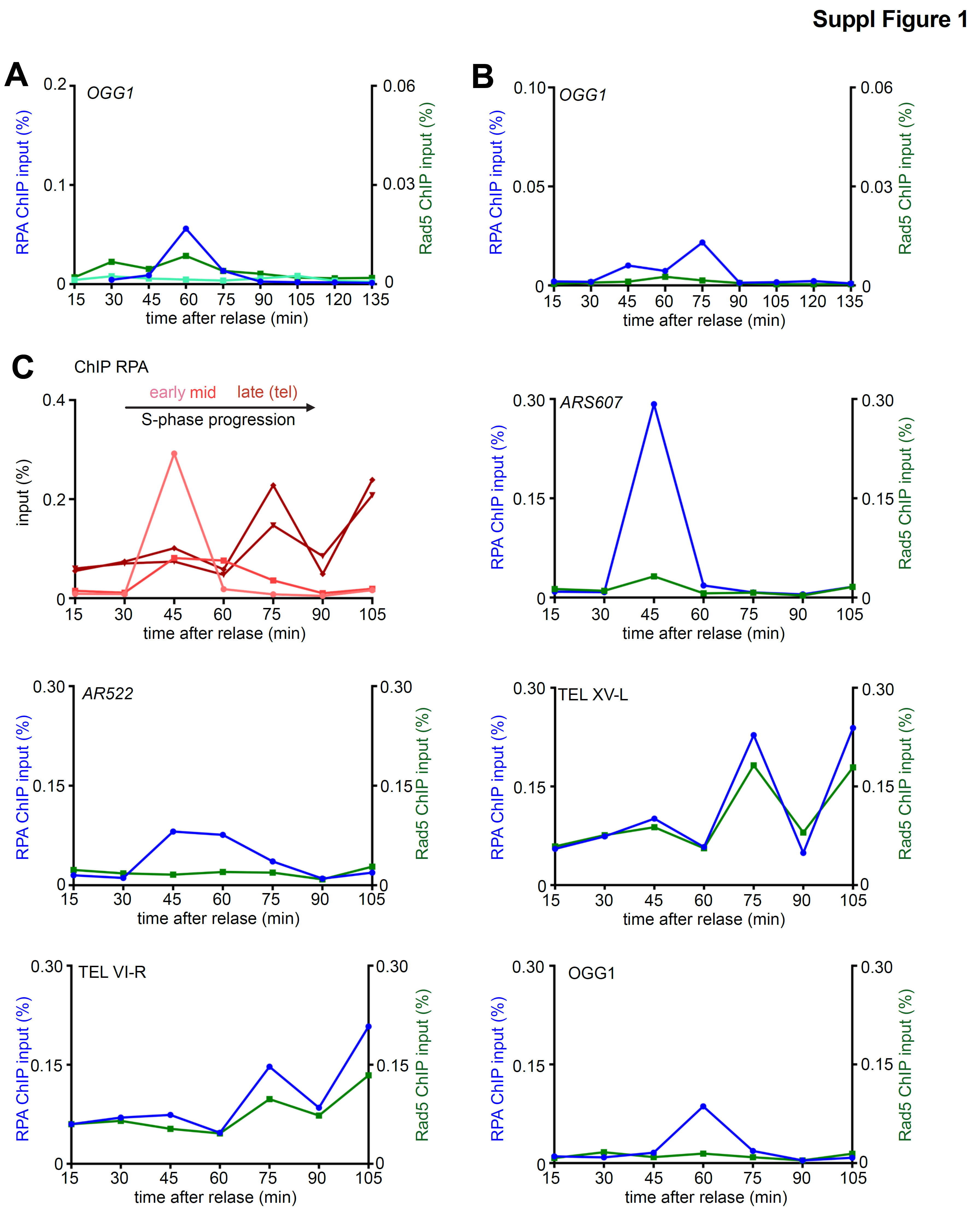


**Supplementary Figure 1. Rad5 is enriched at replicating telomeres. (A-B)**. The ChIP samples used in **Figure 1** were analyzed to assess RPA and Rad5-Myc enrichment at the *OGG1* locus, an additional control for the Rad5-Myc ChIP. The replicative origin closest to the *OGG1* locus is the middle/late firing origin *ARS1307.5*. In the RPA ChIP, DNA replication fork passage is evident at 60 and 75 minutes. (**C).** Triplicate experiment corresponding to **Figure 1**. In this replicate, cells were released into S phase at 19°C following Pronase addition.
